# The daily rhythm of milk synthesis is dependent on the timing of feed intake in dairy cows

**DOI:** 10.14814/phy2.12049

**Published:** 2014-06-24

**Authors:** L. Whitney Rottman, Yun Ying, Kan Zhou, Paul A. Bartell, Kevin J. Harvatine

**Affiliations:** 1 Department of Animal Science, Penn State University, University Park, Pennsylvania; 2 Virginia Department of Conservation and Recreation, Division of Soil and Water Conservation, Virginia Polytechnic Institute and State University, Blacksburg, Virginia; 3 Mississippi State University, Starkville, 39762, Mississippi

**Keywords:** circadian rhythm, diurnal pattern, food entrainment, milk synthesis

## Abstract

Regulation of the daily rhythm of milk synthesis is important to production animals and breastfeeding, but is difficult to observe in nursing animals. The rate of food intake varies over the day and is expected to create a daily rhythm of nutrient absorption. The objective of this study was to determine if the timing of food intake entrains a daily pattern of milk synthesis. Seventeen Holstein cows were used in a crossover design. Treatments were ad libitum feeding of a total mixed ration once daily (1× fed) or fed in four equal meals every 6 h (4× fed). Cows were milked every 6 h the last 7 days of each period. There was a treatment by time of day interaction for milk and milk component yield and concentration. Milk fat and protein concentration and yield exhibited a daily rhythm and the amplitude of the rhythm was reduced in 4× fed. In addition, milk fat percent was higher in 4× fed than 1× fed at three of the four milking intervals (0.22–0.45% higher) and 4× fed increased daily milk fat yield. Treatment by time of day interactions were detected for plasma glucose, insulin, and blood urea nitrogen. These variables also fit a cosine function with a 24 h period and the amplitudes of plasma glucose, insulin, and blood urea nitrogen rhythms were decreased by 4× feeding. In conclusion, there is a circadian pattern of milk synthesis in the dairy cow that is responsive to the timing of food intake.

## Abstract (precis)

Regulation of the daily rhythm of milk synthesis is important to production animals and breastfeeding. The objective of this study was to determine if the timing of food intake entrains a daily pattern of milk synthesis. A circadian pattern of milk synthesis was observed that was responsive to the timing of food intake.

## Introduction

Many behavioral activities and physiological processes have a daily rhythm with a 24 h period (See review by Reppert and Weaver 2002). These daily rhythms are adaptive as they anticipate food availability and synchronize nutrient absorption with metabolism (Panda et al. 2002). Daily rhythms can be entrained to environmental cues and are referred to as circadian rhythms if they persist in the absence of environmental cues. A master circadian clock is located in the suprachiasmatic nucleus (SCN) of the brain, but more recent research has described circadian timekeeping mechanisms in peripheral tissues including the mammary gland (Reviewed by Casey and Plaut (2012)). Light is a strong entrainer of the SCN clock, but the timing of feeding also entrains metabolic and behavioral rhythms and peripheral rhythms are especially responsive to entrainment by feeding (Damiola et al. 2000). Interestingly, the timing of food intake can alter the synchronization between the master timekeeper in the SCN and peripheral clocks, resulting in development of numerous disorders including obesity, insulin resistance, and metabolic diseases (Takahashi et al. 2008). For example, nocturnal mice fed only during the light phase of the day invert rhythms of activity and many physiological processes and, in some instances, become obese and insulin resistant (Honma et al. 1983).

A circadian pattern of milk fat synthesis related to the timing of meals is described in breastfeeding women (Prentice et al. 1981; Stafford et al. 1994) and rhythmic expression of the core circadian regulators was observed in RNA extracted from breast milk (Maningat et al. 2009). However, the factors entraining the rhythms and the mechanism of entrainment are not well described. The dairy cow has a well‐recognized natural daily pattern of feed intake and milk synthesis. However, these rhythms are poorly quantified and their entrainment by feeding has not been specifically investigated. A clear diurnal pattern of intake is observed in cows fed total mixed rations (DeVries et al. 2005) and grazing behavior is normally described as crepuscular, with high intake periods occurring at dawn and dusk (Albright 1993). The daily pattern of milk synthesis is also not well described, but is well appreciated by dairy producers and researchers. Quist et al. (2008) reported changes in milk yield and milk fat and protein concentration in commercial herds milked two and three times per day and van der Iest and Hillerton (1989) showed a distinct pattern of milk synthesis in cows milked every 4 h for 2 days.

Milk yield at each milking is a direct measurement of milk and milk component synthesis over the preceding milking interval and thus represents the rhythm of milk synthesis in the mammary gland. The hypothesis of the current experiment was that there is a daily rhythm of milk synthesis that is dependent on the daily pattern of nutrient absorption. Our objective was to characterize the pattern of milk composition and yield of milk components and demonstrate that it is responsive to the timing of feed intake. To achieve this we observed milk synthesis by milking cows every 6 h when fed either one time per day or in equal feedings every 6 h. Dairy cows can be efficiently milked at a high frequency providing a robust model for this investigation. A daily rhythm of milk and milk component synthesis that is partly dependent on the timing of food intake was observed.

## Materials and Methods

### Animals and experimental design

Animal care and all procedures were approved by the Pennsylvania State University Institutional Animal Care and Use Committee. Twenty multiparous Holstein cows (115 ± 41.4 DIM, mean ± SD; Range 70–195 DIM; Parity: 3 = 3^rd^, 2 = 4^th^, 15 = 2^nd^) from the Pennsylvania State University dairy herd were housed in tie stalls and randomly assigned to treatment sequence in a crossover design with 21 day periods. Three cows were removed from the experiment due to mastitis and respiratory disease (1 in the 4× to 1× and 2 in the 1× to 4× group). Manually controlled lights were turned on at approximately 0430 h and off at approximately 2400 h as normal for the management of the herd. All cows received rbST (Posilac; Elanco Animal Health, Greenfield, IN) administered every 14 days.

**Figure 1.**
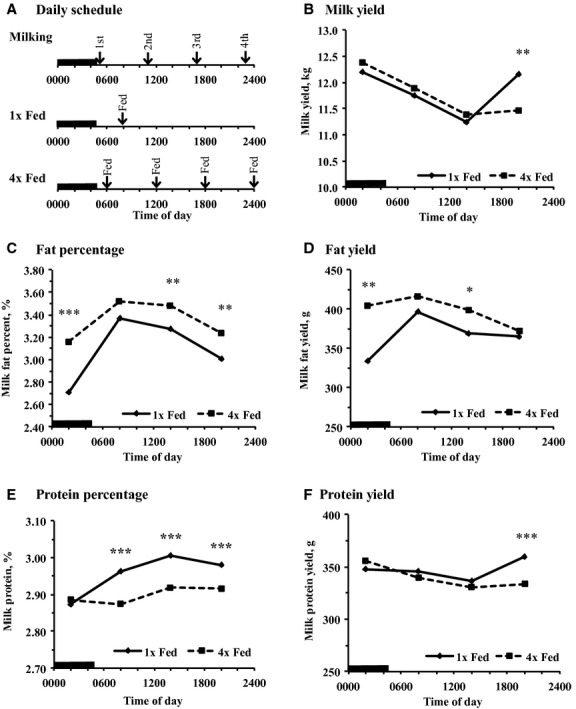
Temporal pattern of milk yield and milk fat and protein yield and percent in cows fed once per day (1× Fed) or in four equal meals every 6 h (4× Fed). Cows were milked every 6 h and data are plotted at the median of the milking interval (MI). (A) Illustration of the timing of milking and feeding, (B) Milk yield (Treatment × Time *P* = 0.05; SEM = 0.42), (C) Milk fat percent (Treatment × Time *P* < 0.05; SEM = 0.15), (D) Milk fat yield (Treatment × Time *P* = 0.06; SEM = 23), (E) Milk protein percent (Treatment × Time *P* < 0.001; SEM = 0.03), (F) Milk protein yield (Treatment × Time *P* < 0.05; SEM = 12). Preplanned contrasts tested the effect of treatment at each milking (*P < 0.05, **P < 0.01, and ***P < 0.001). *n* = 17 per treatment. The black bar indicates the dark phase of the day.

Cows were fed the same total mixed ration (TMR; Table 1) either once a day (1× fed) or in four equal feedings (4× fed). The 1× fed cows were fed at 0800 h at approximately 105% of expected intake and the 4× fed cows were fed equal amounts at 0600, 1200, 1800, and 2400 h at approximately 105% of the previous days total intake (Fig. 1A). All TMR was mixed at 0800 h, manually compacted into plastic barrels, and stored at ambient temperature. Refused feed was removed before delivery of new feed at each feeding. All cows were milked at 0500 and 1700 h from day 1 to 14 of each period (2×/day) and at 0500, 1100, 1700, and 2300 h from day 15 to 21 (4×/day) of each period (Fig. 1A). Data from 4×/day milking are plotted at the median of the milking interval (MI) as each milking represents milk synthesis as the previous milking (e.g., milk collected at 0500 h was synthesized from 2300 to 0500 h and is plotted at 0200 h; Fig. 1).

**Table 1.** Ingredients and nutritional composition of the experimental diet.

Item	Concentration
Ingredients, % DM	
Corn silage	42.3
Alfalfa haylage	13.3
Ground corn	10.8
Soybeans	7.5
Canola meal	6.6
Cookie meal	7.4
Sugar	4.2
Grass/Straw Hay	4.4
Min‐Vit Mix^1^	3.1
NPN^2^	0.4
Composition, % DM^3^	
NDF	31.8
ADF	21.0
CP	16.1
NFC	42.3
Ash	7.1

^1^ Contained (%, as fed basis): 45.8 dried corn distillers grains with solubles; 35.8 limestone (38% Ca); 8.3 magnesium oxide (54% Mg); 6.4 salt; 1.73 vitamin ADE premix; 1.09 selenium premix (0.06% selenium); and 0.88 trace mineral mix. Composition (DM basis): 11% CP; 18% NDF; 5.2% fat; 14.9% Ca; 0.35% P; 4.58% Mg; 0.41% K; 0.31% S; 357 mg/kg Cu; 1,085 mg/kg Zn; 181 mg/kg Fe; 6.67 mg/kg Se; 125,875 IU/kg vitamin A (retinyl acetate); 31,418 IU/kg vitamin D (Activated 7‐dehydrocholesterol); and 946 IU/kg vitamin E (dl‐alpha tocopheryl acetate).

^2^ NPN = slow release nonprotein N fed as Optigen [Alltech Inc. Nicholasville, KY (243% CP, DM basis)].

^3^ Analyzed by Cumberland Valley Analytical Services (Maugansville, MD; *n* = 2). NDF, neutral detergent fiber; ADF, acid detergent fiber; CP, crude protein; NFC, nonfiber carbohydrates [100 − (CP + NDF + EE + ash − neutral detergent insoluble protein)].

### Milk and feed sampling

On day 10–14 and day 18–21 of each period, milk samples were collected at each milking and stored at 4°C with preservative (Bronolab‐WII; D&F Control Systems, Inc., Dublin, CA) until analyzed for fat (Filter B) and true protein using infrared spectrophotometry [Dairy One DHIA, State College, PA; Fossomatic 400; Foss Electric, Hillerød, Denmark; (AOAC 2000)]. An additional milk sample was collected at each milking on day 21 and stored at −20°C until analyzed for fatty acid (FA) profile as described by Rico and Harvatine (2013). The milk FA desaturase indexes were calculated for each sample as an estimation of the activity of the stearoyl Co‐A desaturase enzyme [product / (substrate + product)].

Samples of TMR were collected twice per period using the quartering method and stored at −20°C. Feed samples were dried at 55°C in a forced air oven, ground in a Wiley mill through a 1‐mm screen (Arthur A. Thomas Co., Philadelphia, PA), and analyzed for neutral detergent fiber (NDF), acid detergent fiber (ADF), crude protein (CP), and ash by wet chemistry methods [Described by (Rico and Harvatine 2013); Cumberland Valley Analytical Services Hagerstown, MD].

### Plasma metabolites and hormones

Blood samples were collected six times from day 19 through 21 of each period from the tail vein using potassium EDTA vacuum tubes to represent every 4 h across the day (0200, 0700, 1000, 1400, 1800, and 2200 h). The 0200 h sample was collected on the last day of each period 3 h before the final milk sample. Blood was immediately placed on ice, centrifuged within 1 h, and plasma stored at −20°C until further analysis. Plasma samples were analyzed for insulin (Coat‐a‐count insulin kit; Siemens Healthcare Diagnostics, Los Angeles, CA), glucose [PGO Enzyme procedure no. P 7119; Sigma‐Aldrich, St. Louis, MO (Raabo and Terkildsen 1960)], blood urea nitrogen [BUN; Modified Enzymatic Urea Nitrogen (Procedure No. 2050); Stanbio Laboratory, Boerne, TX], and nonesterified fatty acids [Wako HR Series NEFA‐HR kit (Wako Chemicals USA Inc., Richmond, VA) as modified by Ballou et al. (2009)].

### ANOVA analysis

Milk production, fatty acid, and plasma variables were analyzed using the MIXED procedure of SAS with repeated measures (version 9.2, SAS Institute, Cary, NC). Fixed effects were treatment, time of day, and the interaction of treatment and time of day, and random effects were cow and period. The ARH (1) or AR(1) covariance structures were used based on model fit, the repeated variable was day by time, and subject was cow by period. Denominator degrees of freedom were adjusted by the Kenward–Rogers method. Preplanned contrasts were the effect of treatment at each time point. Daily dry matter intake (DMI) was analyzed without the repeated statement using a reduced model with treatment as the fixed effect and cow and period as random effects. Significance and tendencies of main effects and preplanned contrasts were declared at *P *<**0.05 and *P *<**0.10, respectively, and significance and tendencies of interactions were declared at *P *<**0.10 and *P *<**0.15, respectively.

### Cosine analysis

Time course data were fit to the linear form of a cosine function according to (Bourdon et al. 1995) using random regression in SAS (Seltman 1997). Briefly, the model tested the random effects of sequence, period, and cow and the fixed effects of treatment and the interaction of treatment with the linear form of cosine functions with 24 and 12 h periods. The 12 h harmonic was removed based on significance of cosine model effects and improvement in model fit based on the Bayesian information criterion (BIC). A zero amplitude test was conducted as a *F*‐Test comparing the full to a reduced model to determine the significance of the 24 h cosine fit for each treatment. Phase is reported as acrophase, the time at which the peak of a rhythm occurs. The 95% confidence limits were determined for amplitude and phase (Bourdon et al. 1995) and treatment differences were defined as a difference of amplitude or phase greater than 1.96 times the square root of sum of squares of the standard errors [*P *<**0.05; (Knezevic 2008)]. In all analyses, data points with Studentized residuals outside of ±3 were considered outliers and removed from the analysis. Rarely more than three data points per variable were removed.

## Results

### Dry matter intake

**Figure 2.**
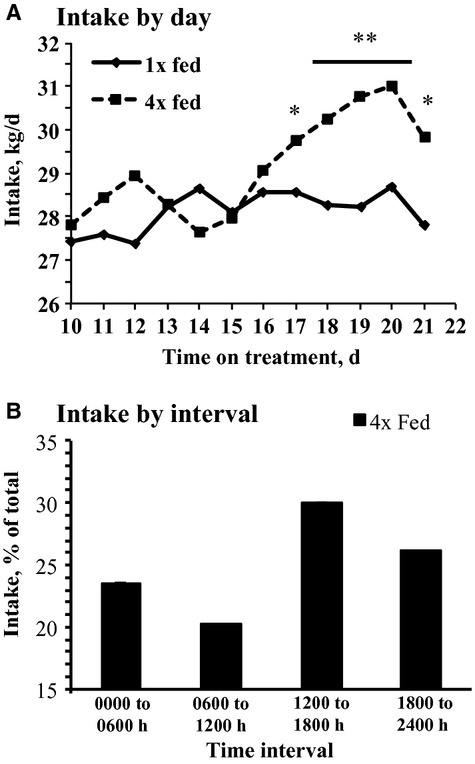
Feed intake in cows fed 1× per day or 4× per day in equal meals. Cows were milked every 12 h from day 1 to 14 and every 6 h from day 15 to 21. (A) Daily dry matter intake from day 10 to 21. Preplanned contrasts tested the effect of treatment at each milking (*P < 0.05 and **P < 0.01; SEM = 1.1–2.2). Panel B: Data are reported as the percent of total intake consumed during each 6 h feeding interval [Time = *P < *0.01; All times differ from each other (*P < *0.01)]. *n* = 17 per treatment.

Cows were fed adequately to allow refusals over the course of the day, but the timing of feed availability was controlled in the 4× fed treatment. There was no effect of treatment on DMI when cows were milked 2×/day (*P *=**0.54; Fig. 2), but 4× fed increased DMI 2.2 kg/day when cows were milked 4×/day during the last 7 day of each period (day 15–21; *P *<**0.01; Table 2; Fig. 2). Differences in feed refusals over the day in the 4× fed treatment resulted in differences in the rate of intake between feeding intervals, with the second interval of the day (0600–1200) having the lowest and the third interval of the day (1200–1800) having the highest rate of feed intake (3.4 and 5.0% of daily intake per h, respectively; Fig. 2).

**Table 2.** Daily milk production and feed intakes in cows fed once per day or in four equal meals every 6 h when milked every 6 h.

Parameter	Treatment^1^		SEM	*P*‐values^2^		
	1× Fed	4× Fed		Trt	Time	Trt × Time
Milk Yield, kg	47.3	47.1	1.5	0.64	<0.001	0.05
Fat, %	3.09	3.35	0.15	<0.001	<0.001	<0.05
Fat Yield, g/day	1465	1592	90	<0.001	<0.01	0.05
Protein, %	2.96	2.90	0.03	<0.001	<0.001	<0.001
Protein Yield, g/day	1389	1360	45	0.06	<0.01	<0.05
DMI, kg	28.2	30.4	1.0	<0.01	‐	‐

^1^ LS means for the treatment of cows fed once a day (1× Fed) or in four equal meals every 6 h (4× Fed). *n* = 17 per treatment.

^2^ Main effect of treatment (trt) and milking time (time) and the interaction of treatment and milking time.

### Milk and milk component yield

Milk yield at each milking is a direct measurement of milk and milk component synthesis over the preceding milking interval and thus represents the rhythm of milk synthesis in the mammary gland. There was no main effect of treatment on total milk yield during either 2×/day or 4×/day milking (Data not shown). There was a treatment by time of day interaction for milk yield during 4×/day milking (*P *=**0.05; Fig. 1A). Milk yield was highest for both treatments during the first MI and decreased during the second and third MI. In the fourth MI, 1× fed milk yield was 0.7 kg higher than 4× fed (*P* < 0.01). Milk yield over the day fit a cosine function with a 24 h period (Table 4). The amplitude of the rhythm of milk yield was not different between treatments, but the phase was delayed by 3.2 h in 4× fed (*P *<**0.05).

Milk fat is the milk component most responsive to nutritional and environmental factors. There was a treatment by time of day interaction for milk fat concentration and yield (Table 2). Milk fat concentration was lowest at the first MI, increased during the second and third MI, and finally decreased during the fourth MI in both treatments (Fig. 1C), but 4× fed cows had higher fat concentration at three of the four milkings compared with 1× fed cows (1^st^, 2^nd^, and 3^rd^ milking; *P *<**0.01). Milk fat yield followed a similar daily pattern, except the second highest milk fat yield was at the first MI with 4× feeding. The 4× fed was also higher than 1× fed at the first MI and third milking intervals (Fig. 1D). Milk fat concentration and yield fit a cosine function with a 24 h period in both treatments (Table 4). Milk fat percent was phase advanced by 1.3 h, whereas milk fat yield was phase delayed by nearly 9 h in 4× fed compared to 1× fed. The amplitude of milk fat concentration was over 40% greater in 1× than 4× fed, but the amplitude of milk fat yield did not differ. Overall, daily milk fat yield was 127 g higher and milk fat percent was 0.26 percentage units higher for 4× fed (Table 2). Similarly, during 2×/day milking, 4× fed had higher milk fat concentration and yield than 1× fed (*P *<**0.001; Data not shown).

Milk protein is also responsive to nutrition and environmental factors, but the magnitude of response is normally smaller. There was a treatment by time of day interaction for milk protein percent with no difference between treatments during the first MI, but 1× fed cows had higher milk protein concentration in the second, third, and fourth MI (Fig. 1E). There also was a treatment by time of day interaction for protein yield. There was no effect of treatments during the first three MI, but a 25 g increase for 1× fed during the fourth MI. Milk protein concentration and yield fit a cosine function with a 24 h period in both treatments and milk protein concentration and yield were phase delayed in 4× fed (2.5 and 4.7 h, respectively; Table 4). The amplitude of milk protein concentration was reduced by 4× fed, but the amplitude of protein yield was increased. The overall daily weighted average milk protein concentration was 0.06% units higher in 1× fed cows (*P *<**0.001; Table 2), but daily milk protein yield only tended to be increased (*P *=**0.06). Similarly, during 2×/day milking, 1× fed also had higher milk protein concentration compared with 4× fed (2.99 and 2.93%, respectively) and milk protein yield did not differ between treatments (Data not shown).

### Milk FA profile

**Figure 3.**
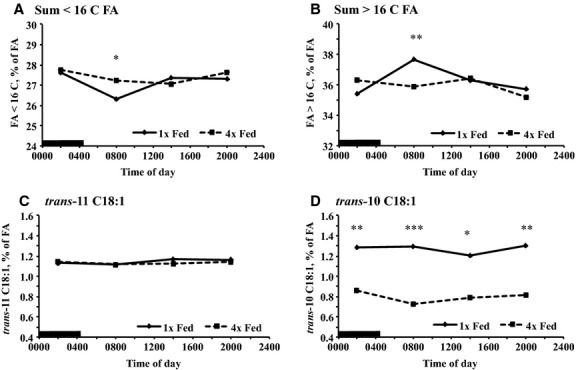
Temporal pattern of concentrations of de novo and preformed fatty acids and select intermediates of the normal (*trans‐*11 C18:1) and alternate (*trans‐*10 C18:1) biohydrogenation pathways in milk fat of cows fed once per day (1× Fed) or in four equal meals every 6 h (4× Fed). Cows were milked every 6 h and data are plotted at the median of the milking interval (MI). (A) Sum of fatty acids less than 16 carbons (De novo synthesized; Treatment × Time *P *<**0.01; SEM = 0.68), (B) Fatty acids greater than 16 C (Preformed fatty acids; Treatment × Time *P *<**0.001; SEM = 0.49), (C) *trans‐*11 C18:1 (Treatment *P *=**0.78, Time *P *=**0.25, Treatment × Time *P *=**0.30; SEM = 0.06), (D) *trans‐*10 C18:1 (Treatment *P *<**0.01, Time *P *=**0.50, Treatment × Time *P *=**0.13; SEM = 0.31). Preplanned contrasts tested the effect of treatment at each milking (*P <**0.05, **P <**0.01, and ***P <**0.001). *n* = 17 per treatment. The black bar indicates the dark phase of the day.

Milk fatty acid profile provides insight into the source of FA and rumen FA biohydrogenation capacity and pathways. There was a treatment by time interaction for the concentration of FA less than 16 carbons (de novo synthesized), 16 carbon FA, and FA greater than 16 C (preformed FA; Table 3). There were no differences between treatments for these FA categories during the first, third, and fourth MI. However, during the second MI, 4× fed had a higher concentration of de novo synthesized and 16 carbon FA (*P *<**0.05; Fig. 3A; Data not shown) and a lower concentration of preformed fatty acids (*P *<**0.01; Fig. 3B). In addition, de novo and preformed FA concentration both fit a cosine function with a 24 h period in 1× fed, but not 4× fed (Table 4). The concentration of individual FA generally followed the pattern of their category (Data not shown). There was no treatment by time of day or time of day effect on the yield of de novo, 16 carbon, and preformed FA (Table 3).

**Table 3.** Milk fatty acid profile of cows fed once per day or in four equal meals every 6 h.

	Treatment^1^		SEM	*P*‐value^2^		
	1× Fed	4× Fed		Trt	Time	Trt × Time
Fatty acid concentration						
* Trans* FA^3^	g/100 g FA					
* trans*‐10 18:1	1.27	0.80	0.31	<0.01	0.50	0.13
* trans*‐11 18:1	1.15	1.13	0.06	0.78	0.25	0.30
**FA by source^4^	g/100 g FA					
**∑ < 16 Carbons	36.27	35.95	0.68	0.41	0.020	<0.01
**∑ 16 Carbons	29.67	30.09	0.59	0.17	<0.01	<0.001
**∑ > 16 Carbons	27.16	27.43	0.49	0.36	<0.001	<0.001
Fatty acid yield						
* Trans* FA	g/day					
* trans*‐10 18:1	8.00	5.90	0.99	<0.01	0.32	0.02
* trans*‐11 18:1	8.30	8.98	1.12	0.27	0.68	0.48
**FA by source	g/day					
**∑ < 16 Carbons	193	213	17	0.05	0.20	0.74
**∑ 16 Carbons	212	237	21	0.03	0.15	0.72
**∑ > 16 Carbons	252	269	24	0.21	0.33	0.17
**Desaturase Activity^5^	Ratio					
**C14 Index	0.098	0.090	0.010	0.16	0.15	0.09
**C16 Index	0.052	0.048	0.004	0.20	0.33	<0.01
**CLA Index	0.377	0.356	0.017	0.07	<0.01	<0.001

^1^ LS means for the treatment of cows fed once a day (1× Fed) or in four equal meals every 6 h (4× Fed). *n* = 17 per treatment.

^2^ Main effect of treatment (trt) and milking time (time) and the interaction of treatment and milking time.

^3^ FA, fatty acid; LA, linoleic acid; ALA, alpha linolenic Acid.

^4^ Fatty acids <16 carbons originate from mammary de novo synthesis, fatty acids >16 carbons originate from extraction from plasma, and 16 carbon fatty acids originate from both sources.

^5^ C14 index = C14:1 / (C14:0 + C14:1), C16 Index = C16:1 / (C16:0 + C16:1), CLA index = *cis‐*9, *trans‐*11 C18:2 / (*trans‐*11 C18:1 + *cis‐*9, *trans‐*11 C18:2).

**Table 4.** The phase and amplitude of a cosine function with a 24 h period fit to milk yield and composition, milk fatty acid profile, and select plasma metabolites and hormones of cows fed once per day or in four equal meals when milked every 6 h.

Parameter	Trt^1^	Phase^2^ (h)	Amplitude^3^	*P‐*value^4^
Milk production				
Milk yield, kg/6 h	1× Fed	0336	0.55	<0.001
	4× Fed	0648*	0.50	<0.001
Fat, %	1× Fed	1500	0.34	<0.001
	4× Fed	1342*	0.24*	<0.001
Fat yield, g/6 h	1× Fed	01430	21	<0.01
	4× Fed	2324*	22	0.02
Protein, %	1× Fed	1718	0.06	<0.001
	4× Fed	1948*	0.03*	<0.001
Protein yield, g/6 h	1× Fed	0148	9.1	<0.05
	4× Fed	0630*	14*	<0.001
Milk FA by source				
∑ < 16 C,% FA	1× Fed	2350	0.91	<0.001
	4× Fed	0520*	0.28*	0.29
∑ 16 C,% FA	1× Fed	2338	0.61	<0.01
	4× Fed	0242*	0.43*	0.01
∑ > 16 C,% FA	1× Fed	1235	1.09	<0.001
	4× Fed	1133	0.25*	0.50
Plasma hormones and metabolites				
Glucose, mg/dL	1× Fed	0048	2.5	<0.001
	4× Fed	0912*	0.68*	0.35
Insulin, *μ*IU/mL	1× Fed	1736	8.0	<0.001
	4× Fed	1542*	2.7*	<0.001
NEFA, *μ*Eq/L	1× Fed	0530	21.2	<0.001
	4× Fed	700*	7.7*	0.12
BUN, mg/dL	1× Fed	1000	1.2	<0.001
	4× Fed	1130*	0.39*	0.10

^1^ Treatments (Trt) were cows fed once a day (1× Fed) or in four equal meals every 6 h (4× Fed). *n* = 17 per treatment.

^2^ Phase is the time (h) of peak of a cosine function with a 24 h period. * Indicates 4× Fed different from 1× Fed (*P *<**0.05).

^3^ Amplidute is the difference from peak to mesor of a cosine function with a 24 h period. Units shown next to parameter.

^4^ Zero amplitude test of the cosine function with a 24 h period for each treatment.

The majority of 18 carbon FA in milk originate directly from nutrient absorption providing a sensitive indication of absorbed FA profile. In addition, *trans‐*10 18:1 is a major intermediated of the alternate rumen biohydrogenation pathways that predominate during diet‐induced milk fat depression whereas *trans‐*11 18:1 is a major intermediate of the normal biohydrogenation pathway. There was a main effect of treatment on milk fat *trans‐*10 18:1 (*P *<**0.01), but only a tendency for a treatment by time of day interaction (*P *=**0.13; Table 3). Milk *trans‐*10 18:1 was at least 0.40% units higher in 1× fed than 4× fed at all milkings (*P *<**0.05; Fig. 3D). There was a treatment by time of day interaction for *trans‐*10 18:1 yield (*P *=**0.02), with the largest difference at the second MI and the smallest at the third MI. There was no effect of treatment, time of day, or interaction of treatment and time of day for the milk fat *trans‐*11 18:1 concentration or yield (Table 3; Fig. 3C).

The desaturase indexes provide insight into the activity of the delta‐9 desaturase enzyme which is very responsive to nutritional and metabolic signals. There was a treatment by time of day interaction for the C14, C16, and CLA desaturase indexes (Table 3). All indexes were decreased in 4× fed at the second MI (*P *<**0.05), but did not differ between treatments at the other intervals. Lastly, the desaturase indexes failed to fit a cosine function with a 24 h period in all treatments (Data not shown).

### Plasma metabolites and hormones

**Figure 4.**
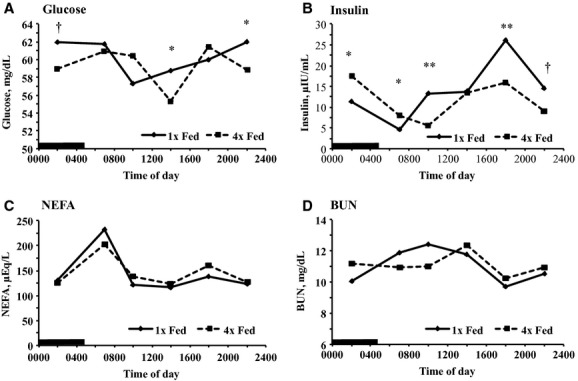
Temporal pattern of the concentration of plasma metabolites and hormones in cows fed once per day (1× Fed) or in four equal meals every 6 h (4× Fed). Cows were milked every 6 h and data are plotted at the median of the milking interval (MI). (A) Plasma glucose (Treatment *P = *0.33, Time *P < *0.001*,* Treatment × Time *P < *0.01; SEM = 1.72), (B) Plasma insulin (Treatment *P = *0.06, Time *P < *0.001*,* Treatment × Time *P < *0.001; SEM = 3.22), (C) Plasma nonesterified fatty acids (NEFA; Treatment *P = *0.81, Time *P < *0.001*,* Treatment × Time *P *=**0.29; SEM = 24), and (D) Plasma blood urea nitrogen (BUN: Treatment *P = *0.92, Time *P < *0.001, Treatment × Time *P < *0.05; SEM = 0.66). Preplanned contrasts tested the effect of treatment at each milking (^†^*P < *0.10, *P < 0.05, and *^*^P < 0.01). *n* = 17 per treatment. The black bar indicates the dark phase of the day.

Plasma metabolites and hormones were measured to provide insight into dynamics in nutrient absorption and endocrine response. There was a treatment by time of day interaction for plasma glucose (*P *<**0.001; Fig. 4A). Plasma glucose peaked between 2200 and 0600 h in 1× fed and fell to a nadir at 1000 h, whereas 4× fed peaked at 0600 and 1800 h and fell to a nadir at 1400 h (Fig. 4A). Plasma glucose tended to be higher in 1× fed at 0200 h and was higher than 4× fed at 1400 and 2200 h. A cosine function with a 24 h period fit plasma glucose in 1× fed, but not 4× fed (failed zero amplitude test). Similar to plasma glucose, there was a treatment by time interaction for plasma insulin concentration (*P *<**0.001), with 1× fed having lower insulin at 0200 and 0700 h (*P *<**0.01) and higher insulin at 1000 and 1800 h compared to 4× fed (*P *<**0.01; Fig. 4B). Insulin concentration fit a cosine function with a 24 h period in both treatments, and insulin was phase advanced (1.9 h) and decreased in amplitude (66%) by 4× fed.

Plasma NEFA is an indication of the rate of FA mobilization from adipose tissue. There was a main effect of time of day, but no effect of treatment or interaction of treatment and time of day on plasma NEFA. Both treatments peaked at 0600 h, decreased immediately afterwards, and remained low for the rest of the day (Fig. 4C). Plasma NEFA fit a cosine function with a 24 h period in 1× fed, but not in 4× fed (Table 4).

Lastly, BUN provides insight into rumen ammonia concentration, absorption of nonprotein nitrogen, and hepatic urea synthesis. There was a treatment by time interaction for BUN (*P *<**0.05), but there were no difference between treatments at any time points (*P* = 0.02; Fig. 4D). Plasma BUN fit a cosine with a 24 h function in the 1× fed, but not the 4× fed (failed zero amplitude test).

## Discussion

Circadian rhythms are entrained and modified by numerous environmental signals and the interaction of these signals and milk synthesis has not been well investigated. Possible entraining cues for the dairy cow are the light/dark cycle and the timing of feeding, milking, and other daily management activities. The current experiment was designed to investigate the effect of the natural pattern of feed intake on the daily rhythms of milk synthesis and key plasma metabolites and hormones. The 1× fed treatment allowed cows to consume feed naturally over the day, which is influenced by the timing of feed delivery and milking (DeVries et al. 2005). Although not measured in the current experiment, a high rate of intake is expected after feeding and during the afternoon (approximately 5–15% of daily intake per h) and a low rate of intake is expected during the overnight period (approximately 1.5–2.5% of daily intake per h) with once per day feeding in the morning (unpublished data). Feeding cows frequently at evenly spaced intervals equalizes intake over the day and intensive experiments seeking establishment of steady‐state conditions will commonly use hourly or bi‐hourly feed delivery. In the current experiment, feeding every 6 h in four equal meals was selected to minimize the disturbance of natural behaviors while distributing intake over the day. Cows were fed in equal meals, but were also fed above expected daily intake, as feed restriction would decrease milk yield. The extent of uneven feed refusals over the day is dependent on the total daily refusals allowed, so care was taken to control total daily refusal without limiting daily intake. Feed was mixed only once per day and the lowest intake was observed after the morning feeding (0600 h) when feed was 22 h old and presumably less palatable, but feed intake was distributed across the day.

Treatments were randomly assigned to stalls creating interspersion of treatments, which may have initially caused cross‐entrainment. However, cows adapted quickly to treatment and little disturbance were observed in cows assigned to the opposite treatment at feedings. In addition, we have observed very little cross‐entrainment in other experiments that similarly housed cows and fed at different times of day while observing feeding behavior using an automated observation system (Niu et al. 2013).

Selective intake, or feed sorting, also changes the amount of digestible nutrients consumed over the day. The 4× fed treatment is expected to have limited the ability of cows to sort feed during the early part of the day. Cows preferentially sort for short particles (Leonardi and Armentano 2003) and the opportunity for sorting is dependent on the amount of feed available. It is reasonable to expect that some of the 1× fed cows consumed not only more feed after feeding, but also feed higher in starch and lower in fiber. Conversely, it is expected that the 1× fed cows consumed feed higher in fiber and lower in fermentable substrate as the day progressed (Leonardi and Armentano 2003). The 4× fed cows would also be expected to sort at each feeding, but the magnitude of sorting would be limited by the smaller amount of feed available. As the objective of the experiment was to compare uneven nutrient intake to more evenly distributed nutrient intake, increased sorting in 1× fed cows would benefit this design by further exacerbating differences in fermentable organic matter intake over the day.

Dry matter intake progressively increased in 4× fed after the initiation of 4×/day milking (Fig. 4), although the experiment was not designed to test the interaction of feeding and milking frequency and the exact reason for this response is not clear. Milking and offering fresh feed stimulate feeding behavior (DeVries et al. 2005) and 4× fed cows received fresh feed after returning from the parlor. If feed delivery at the return from milking increased intake it is expected to be a short‐term effect that would subside over the long‐term. In general, increasing feeding frequency up to five times per day changes feeding pattern by creating more spikes in feeding behavior around the time of each feeding, but does not increase daily feed intake (e.g., Nocek and Braund 1985; DeVries et al. 2005; Mantysaari et al. 2006). Offering fresh feed more than four times per day also increased feed efficiency (Nocek and Braund 1985; Mantysaari et al. 2006) and reduced variation in ruminal pH (French and Kennelly 1990; Shabi et al. 2005). However, Mantysaari et al. (2006) reported increased restlessness and decreased lying time and Phillips and Rind (2001) reported decreased time spent ruminating and disruption of the circadian lying pattern, which must be considered in application of increased feeding frequency.

The daily pattern of milk and component synthesis is not well studied in any species and is limited by twice daily milking commonly used in dairy research experiments and natural nursing in many mammals. In the current experiment cows milked every 6 h provided a higher resolution of milk synthesis over the day. Milking every 4 h has been reported in the literature, but was not selected due to the increased time away from feed and possible confounding influence on natural rhythms. In addition, cows were milked at the increased frequency for only the last week of each period. The first 2 weeks allowed diet adaptation and observation without the influence of increased milking. The cows quickly adapted to the increased milking frequency and no major treatment differences were observed between 2×/day and 4×/day milking.

Dairymen commonly recognized that morning and evening milking differ in milk yield and composition. In a 12 h milking interval, Gilbert et al. (1972) reported an increase of 0.65 kg of milk at the morning milking and an increase in milk fat and protein by 0.32 and 0.09 percentage units, respectively, during the evening milking. In addition, Everett and Wadell (1970) reported that early lactation cows had up to a 1.71 kg higher milk yield at morning milking. More recently Quist et al. (2008) conducted a large survey of the milking‐to‐milking variation in milk yield and composition on 16 dairy farms. Milk yield and milk fat concentration showed a clear repetitive daily pattern over 5 days in herds that were milked 2 and 3×/day. The current experiment demonstrates a daily rhythm of milk and milk fat yield and milk fat and protein concentration in cows milked every 6 h under well‐controlled conditions. Importantly, maintenance of a daily rhythm (e.g., milk fat and protein concentration) with a decreased amplitude in 4× fed cows provides support for partial regulation by the timing of nutrient availability and partial regulation by an endogenous time keeping mechanism.

Circadian regulation in the mammary gland is supported by reports of daily rhythms of mammary enzyme activity and core “clock” genes. For example, lactating mammary tissue in the rat has a daily rhythm of metabolism with approximately a 1‐fold change in lactose synthesis and approximately a 2‐fold change in lipid synthesis (Carrick and Kuhn 1978; Munday and Williamson 1983). In addition, the circadian rhythm of mammary enzymes observed was in synchrony with the daily pattern of intake in the rat. Rhythmic expression of core clock genes was observed in humans (Maningat et al. 2009), and Casey et al. (2009) reported increased expression of BMAL1 and modified expression of six other clock genes on day 1 of lactation compared to day 20 of pregnancy. Lastly, global deletion of the CLOCK gene in mice reduced milk yield (Hoshino et al. 2006), providing evidence of the importance of circadian rhythms in lactation, although the direct functional role of CLOCK in the mammary gland is not clear.

Milk fat synthesis is well known to be responsive to nutrition and specific *trans* fatty acid isomers produced in the rumen decrease milk fat synthesis in the mammary gland by inhibiting enzymes involved in lipid synthesis (Reviewed by Harvatine et al. 2009). Interpretation of the milk fat response to 4× feeding is difficult because of changes in both rumen fermentation and the temporal pattern of nutrient absorption. *Trans‐*10 C18:1 is an indicator of the altered pathway of biohydrogenation associated with diet‐induced milk fat depression (Harvatine et al. 2009) and higher concentrations of this isomer were observed at all milkings in 1× fed in agreement with lower milk fat concentration and decreased fat yield. However, it appears that changes in absorption of biohydrogenation intermediates and transfer to the mammary gland over the day does not explain the rhythm of milk fat synthesis as the concentration of *trans‐*10 C18:1 was nearly constant over the day. Peak transfer of FA into milk is expected by 4 to 6 h after absorption (Harvatine and Bauman 2011), so the 6 h resolution of milking is expected to be adequate to observe differences in *trans‐*isomer absorption over the day, but observation of the response may be diminished by the timing of milking intervals.

Decreased de novo and increased preformed FA in milk at the second MI coincided with a change in the desaturase index and may indicate a modification of mammary lipid metabolism during this MI by bioactive FA or an endogenous rhythm. The desaturase enzyme is highly responsive to nutrition and is increased in some cases of diet‐induced milk fat depression (Rico and Harvatine 2013), but is decreased by *trans*‐10, *cis‐*12 CLA (Reviewed by Harvatine et al. 2009). In addition, de novo FA synthesis is more sensitive to bioactive FA that cause diet‐induced milk fat depression (Harvatine et al. 2009). However, the largest difference in fat yield between treatments occurred at the first MI.

Milk protein concentration and yield showed daily patterns that were responsive to the timing of feed intake. This may be partially explained by the observed changes in insulin, as exogenous administration of insulin increases milk protein presumably through stimulation of IGF‐1 (Griinari et al. 1997). Increased insulin at 1800 h corresponds to the timing of increased milk protein synthesis during the fourth MI of the day and continuing into the first MI. The timing of amino acid absorption may also have an impact on milk protein synthesis. Although it has not been directly measured, duodenal flow of microbial protein would be expected to peak later in the day following peak ruminal fermentation and microbial growth in cows fed once per day in the morning. The ruminant absorbs ammonia through the rumen epithelium and the rate is increased when rumen ammonia increases because the rate of protein degradation is greater than the rate of ammonia use by the microbes. The change in BUN over the day may indicate changes in the amount of protein degraded over the day, presumably due to changes in the rumen degradable protein pool or microbial proteolysis capacity (Reynolds and Kristensen 2008).

Plasma NEFA are commonly observed to peak immediately before feeding in experiments when cows are fed in the morning, which is normally attributed to the daily rhythms of absorbed nutrients and plasma insulin. For example, Nikkhah et al. (2008) reported peak NEFA immediately before feeding in cows fed at 0900 h and peak NEFA 6 to 8 h before feeding in cows fed at 2100 h. In the current experiment NEFA peaked in the morning in both the 1× and 4× fed suggesting that NEFA are regulated by additional mechanisms than the timing of nutrient availability. Plasma NEFA are an indicator of lipid mobilization from adipose tissue and are quickly cleared from the blood making them a sensitive indicator of temporal changes. Therefore, the daily pattern of fat mobilization appears to be explained by its association with the circadian system similar to that observed in rodents (Froy 2011) and in the cow may not be as tightly associated with feeding as commonly assumed.

Changes in plasma glucose indicate either changes in the rate of gluconeogenesis or rate of plasma glucose clearance, as the ruminant absorbs little glucose. The daily pattern of plasma glucose was modified by 4× feeding indicating possible changes in propionate absorption and flux to the liver or circadian regulation of gluconeogenesis and glucose disposal. Increased propionate flux to the liver would increase gluconeogenesis if propionate is limiting the rate of gluconeogensis, but excess propionate would be oxidized if propionate is not limiting (Allen and Bradford 2012). However, it has been recently reported that the pancreas has a functioning clock regulating insulin production, thus providing a link between circadian rhythms and regulation of plasma glucose and gluconeogenesis (Marcheva et al. 2011). The pattern of insulin was modified by feeding schedule, but the changes in insulin do not correspond to the observed changes in plasma glucose.

In conclusion, there is a daily rhythm of milk and milk component synthesis that is partially dependent on the timing of feed intake. Generally, distributing feed intake across the day reduced the amplitude of the rhythm of milk synthesis and plasma metabolites and hormones, which indicates an underlying circadian rhythm partially responsive to nonfeeding factors. Future work investigating changes in the molecular clock in the mammary gland will be essential to definitively understand the mechanism of the daily rhythm of milk synthesis.

## Acknowledgments

The authors gratefully acknowledge the technical assistance of D. Rico (Penn State University, University Park PA). Gratitude is also expressed to the Pennsylvania State University Dairy Cattle Research and Education Center.

## Conflict of interest

None declared.
